# When a Freckle May Not Be a Freckle: A Case of Lentigo Maligna

**DOI:** 10.7759/cureus.94304

**Published:** 2025-10-10

**Authors:** Madeline Tchack, Kiran Javaid, Bassem Rafiq, Noah Musolff, Babar Rao

**Affiliations:** 1 Medical School, Rutgers Robert Wood Johnson Medical School, Piscataway, USA; 2 Molecular Biology, Rowan-Virtua School of Osteopathic Medicine, Stratford, USA; 3 Dermatology, Rao Dermatology, Atlantic Highlands, USA; 4 Dermatology, Robert Wood Johnson University Hospital, Somerset, USA

**Keywords:** dermoscopy, dermoscopy image analysis, general dermatology, lentigo maligna, outpatient family medicine, preventative measures

## Abstract

Lentigo maligna (LM) is a subtype of melanoma that typically arises in chronically sun-exposed skin. A case of LM arising on the right malar cheek of a 51-year-old male patient is presented herein. Clinically, the patient’s lesion appeared benign and lacked overt features of malignancy. Dermoscopic evaluation, however, revealed asymmetric pigmentation concerning for malignancy. A biopsy was subsequently performed, confirming the diagnosis of LM. This case highlights the importance of incorporating dermoscopy into the routine evaluation of pigmented lesions, as reliance on clinical inspection alone may result in misclassification and inappropriate management.

## Introduction

Lentigo maligna (LM) is a subtype of malignant melanoma that most commonly presents as an irregularly pigmented brown macule on chronically sun-exposed areas, such as the head and neck [1]. In contrast, solar lentigines and freckles (sunspots) are common benign lesions that frequently arise in the same locations, making early recognition of LM particularly challenging. This diagnostic overlap underscores the clinical relevance of distinguishing a simple “freckle” from an early LM. 

Clinically, LM often presents as a slowly enlarging, irregular, tan-brown patch with variegated pigmentation and indistinct borders, most frequently occurring on sun-damaged facial skin such as the cheek and nose [1,2]. Histologically, LM is characterized by atypical melanocytes along the basal layer, often extending down adnexal structures, without dermal invasion [2]. 

The primary risk factor for developing LM is cumulative ultraviolet radiation [3]. If left undiagnosed, LM may progress to invasive melanoma, most often of the aggressive desmoplastic subtype, in approximately 5-20% of patients [4]. Epidemiologically, LM and LM melanoma (LMM), a more invasive type of LM, account for over 15% of all melanoma cases in the United States, with incidence rates rising steadily in recent decades [5]. Given this potential for progression, accurate and timely diagnosis is critical. 

Dermoscopy has been shown to increase diagnostic accuracy nearly ninefold compared to unaided clinical examination [6]; however, LM may still masquerade as a benign lesion on first inspection. Our case is distinctive because the patient’s LM closely resembled a benign freckle and was initially reassured as such by a primary care physician (PCP). 

We present this case to highlight how LM can mimic innocuous-appearing pigmented lesions and emphasize the value of dermoscopy in identifying subtle malignant features that may not be apparent on gross clinical examination, underscoring the need for diagnostic vigilance in both dermatologic and primary care settings. 

## Case presentation

**Figure 1:**
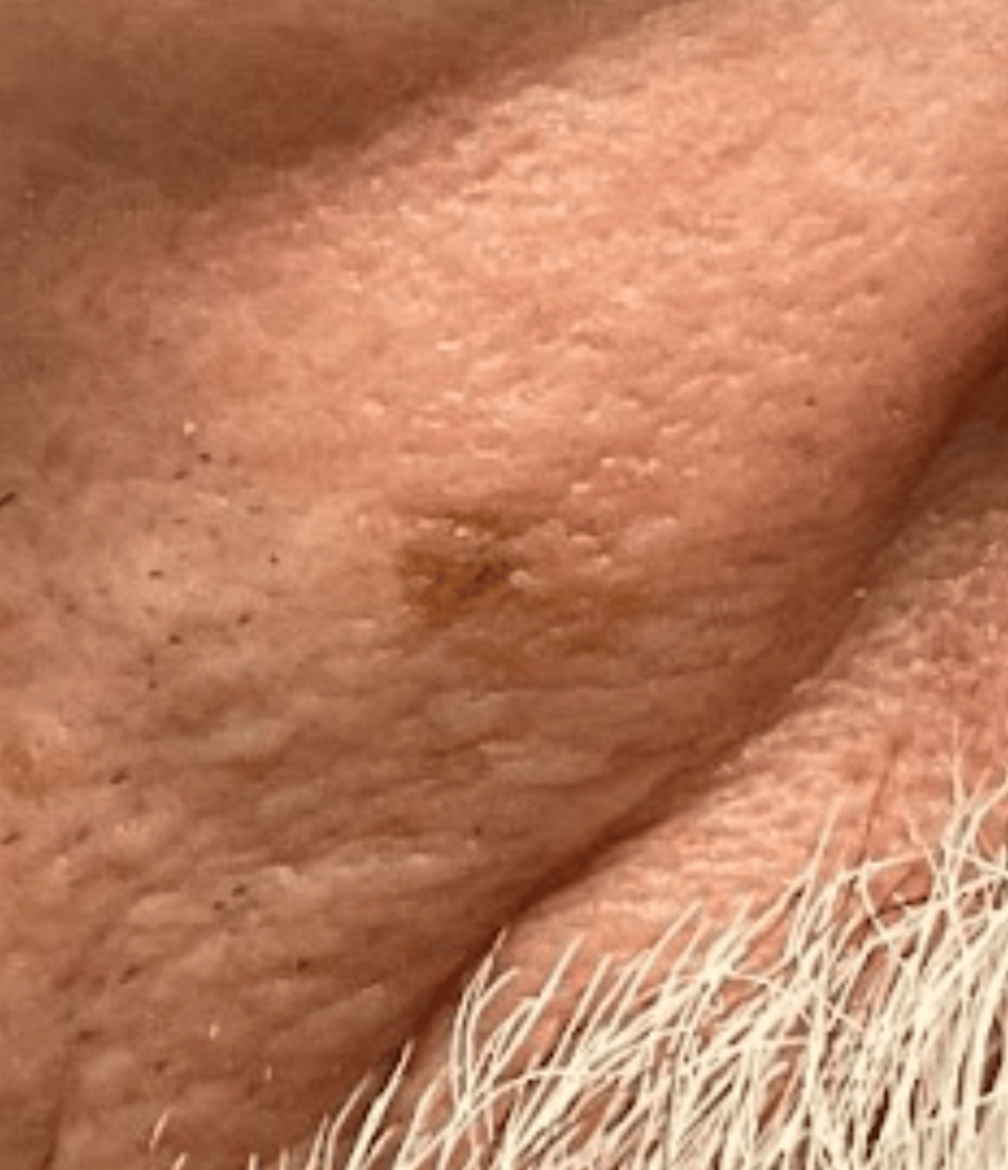
Clinical examination showed a 0.5 x 0.8 cm light brown regular macule on the right cheek.

A 51-year-old male patient presented to the dermatology clinic with a light brown macule on the right cheek (Figure 1) that had been present for several months. On review of systems, the patient revealed no pain, itching, fever, chills, night sweats, or unintended weight loss. He had no personal or family history of melanoma or nonmelanoma skin cancer. His past medical history was significant only for an aortic aneurysm. Of note, the patient had been to his PCP approximately one month prior to this appointment, where he was told the lesion was not a cause for concern. 

**Figure 2:**
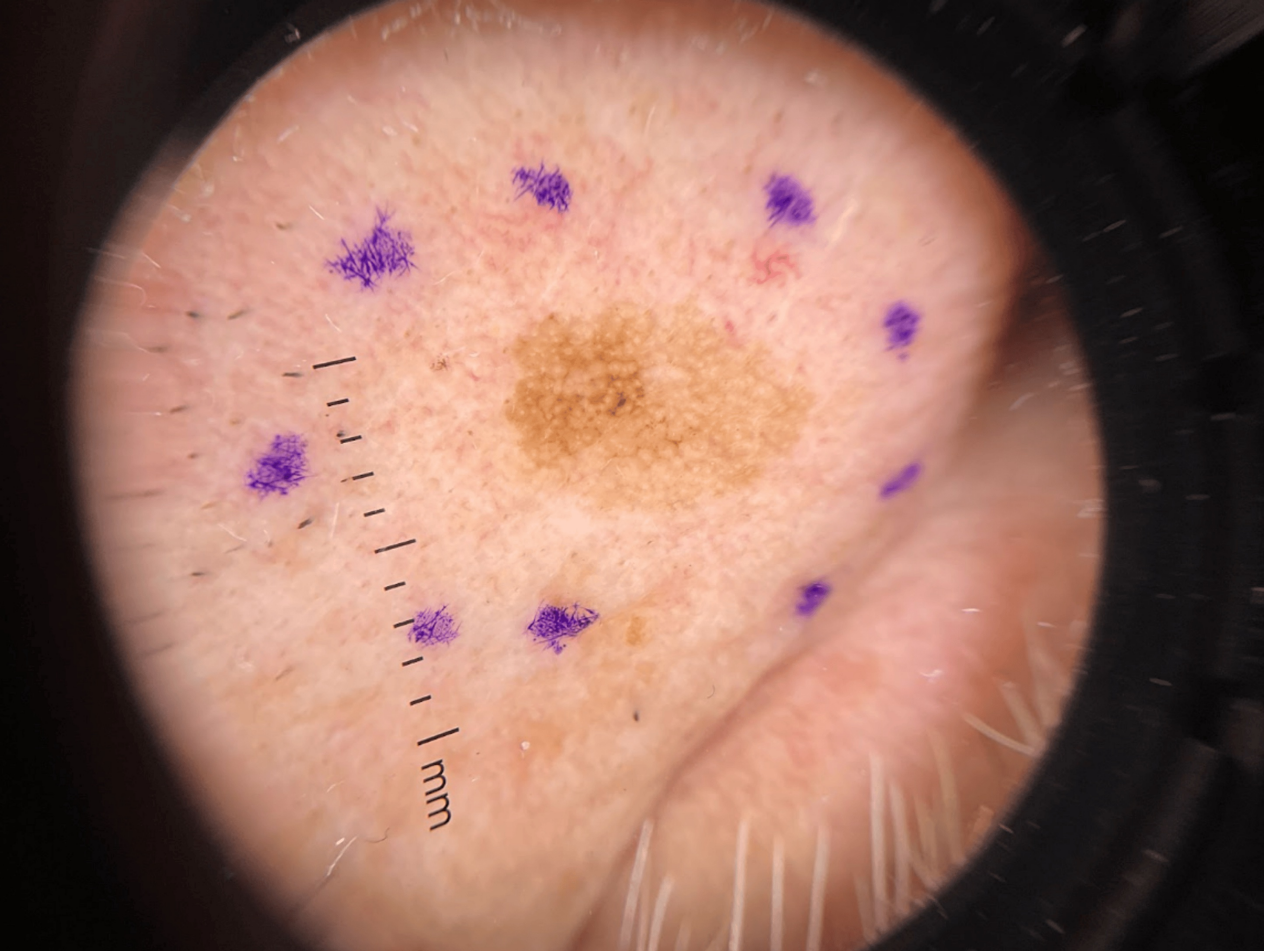
Dermoscopy demonstrated variegated colors, asymmetrical pigmented follicular openings, and angulated lines creating a pseudonetwork with regular borders.

On clinical examination, the lesion did not exhibit features overtly suspicious for malignancy. As part of routine diagnostic practice, dermoscopy was used and showed an asymmetrical pigmented pseudonetwork with regular borders (Figure 2). Given the lesion’s asymmetric pigmentation observed under a dermatoscope, biopsy versus interval monitoring was discussed as part of his management. Ultimately, the patient opted to proceed with a biopsy. 

**Figure 3:**
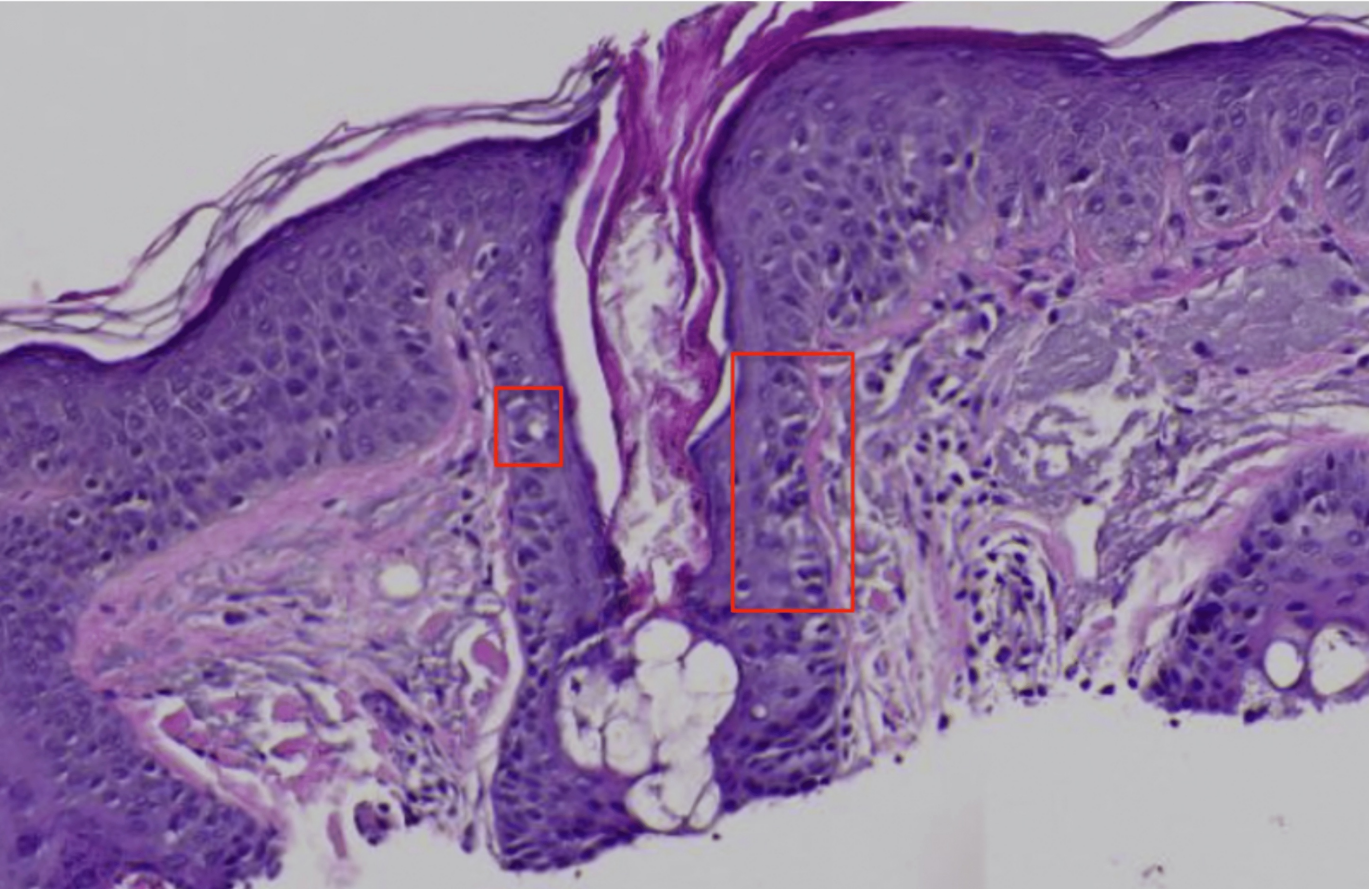
Microphotograph at 400x magnification, stained with H&E. Histopathology showed an increased presence of single and nested pleomorphic cells (red squares) along the dermoepithelial junction including along the hair follicles.

**Figure 4:**
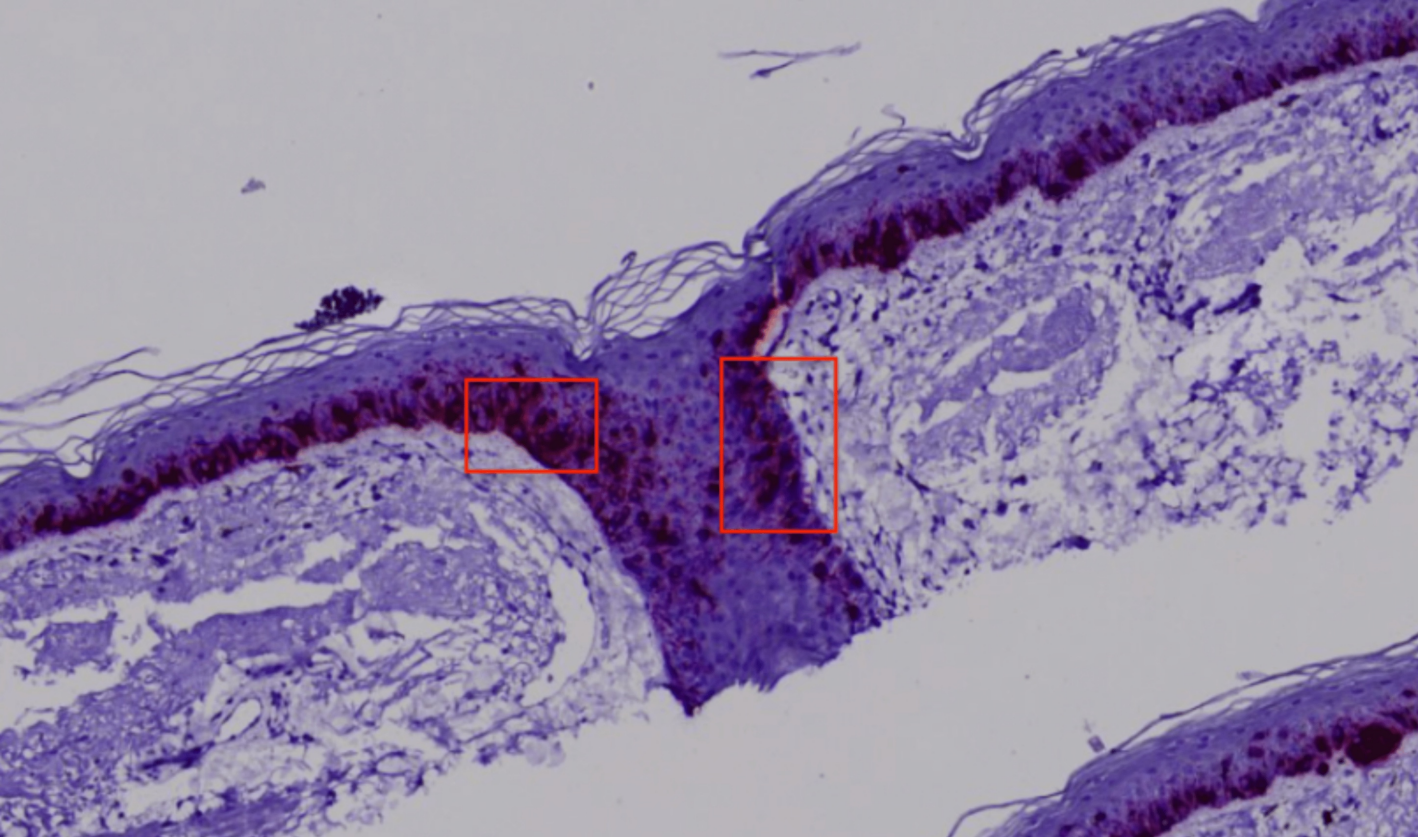
Microphotograph at 400x magnification, immunohistochemically stained with MART-1. The staining revealed an increased uptake of the atypical cells (red squares) at the dermoepithelial junction confirming them to be melanocytes.

**Figure 5:**
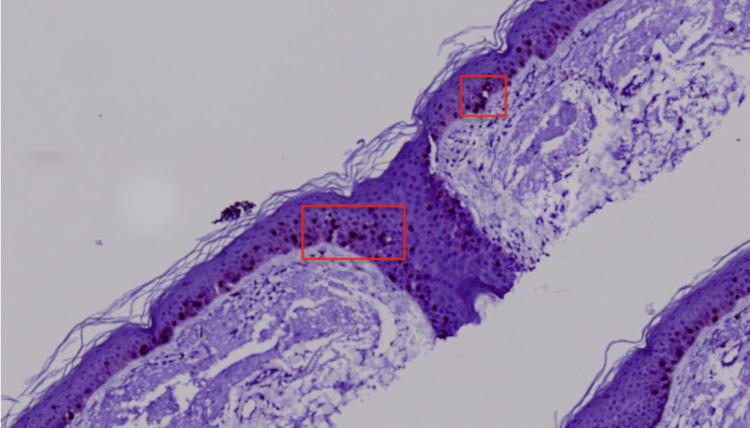
Microphotograph at 400x magnification, immunohistochemically stained with SOX-10. The staining revealed an increased uptake of the atypical cells (red squares) at the dermoepithelial junction confirming them to be melanocytes.

Histopathological examination revealed melanocytes throughout the epidermis in a pagetoid distribution and atypical melanocytes with large hyperchromatic pleomorphic nuclei and abundant cytoplasm along the basal layer of the epidermis extending down appendages with variably sized and shaped melanocytic nests unevenly distributed in the dermal-epidermal junction (DEJ) (Figure 3). Additional staining was performed, demonstrating positive SOX-10 & MART-1 (Figures 4, 5), confirming the diagnosis of melanoma in situ, LM type. Wide local excision was recommended for further management.

## Discussion

Sun-exposed areas are particularly susceptible to ultraviolet radiation, and the cumulative effects of sun damage lead to the development of lentigines, freckles, actinic sun damage, and cutaneous malignancies [7]. While these lesions are most often benign, malignant lesions may mimic those that appear benign, both clinically and on dermoscopy [8]. Important differential diagnoses to consider when evaluating freckles include melanoma, LM, and porokeratosis [9].

Lentigines and freckles are common benign lesions, yet rare cases of progression to melanoma in situ have been reported [10]. In our patient, the lesion lacked the classic ABCDE (asymmetry, border, color, diameter, and evolving) criteria [11] used to clinically identify melanoma, thus appearing clinically benign. Inspection with dermoscopy, however, revealed subtle atypical features, including variegated pigmentation, asymmetrical pigmented follicular openings, and angulated lines creating a pseudonetwork. Dermoscopy of a benign solar lentigo often demonstrates a delicate, light brown typical pseudonetwork with uniform pigmentation, whereas an LM is characterized by atypical pseudonetworks with irregular, asymmetric pigmentation surrounding obliterated or distorted follicular openings [12]. 

This case underscores the importance of incorporating dermoscopy into routine evaluations of new or evolving cutaneous lesions, not only for dermatologists but also for general practitioners. Beyond diagnostic accuracy, dermoscopy can help identify the most diagnostically informative area for biopsy in large or heterogeneous LM lesions, thereby reducing sampling error and improving diagnostic yield [13]. Nevertheless, widespread dermoscopy use in primary care remains limited by barriers such as insufficient training, lack of prioritization for many PCPs, perceived complexity of dermoscopy, and high equipment costs [14]. Despite these challenges, Williams et al. found that most PCPs view dermoscopy as a valuable tool, citing lack of access and training as the primary obstacles, and strongly support its inclusion in residency curricula [15]. Further, a three-month e-learning dermoscopy course for family physicians led to significant diagnostic score improvements in 87.3-92.1% of participants [16], demonstrating that training interventions can be highly effective.

Dermoscopy has consistently been shown to improve skin cancer diagnostic accuracy for dermatologists and PCPs alike [17]. Reliance on clinical examination alone risks misclassifying malignant lesions as benign and improperly managing them with clinical observation. In our case, dermoscopy provided the critical diagnostic cues that prompted biopsy and ultimately revealed LM. This presentation is notable because the lesion was initially misdiagnosed as a benign freckle by a PCP, reflecting the challenge of distinguishing LM from benign pigmented lesions in non-specialist settings.

A limitation of this case is the absence of postoperative follow-up data, including the healing process, aesthetic results, and patient satisfaction. Additionally, detailed information regarding lesion evolution prior to presentation and a formal histologic grading of atypia was not available, which limits further clinicopathologic correlation.

## Conclusions

Overall, our case emphasizes the importance of clinical correlation with dermoscopy, not only in dermatology clinics but also in primary care settings. In the absence of dermoscopy, lesions that appear clinically benign may be misclassified as simple “freckles,” potentially delaying diagnosis and treatment of dangerous lesions. Dermoscopy is necessary for physicians to make informed management decisions, and general practitioners who familiarize themselves with dermoscopic patterns can provide more comprehensive dermatologic care. This message is underscored by our patient, who was initially reassured by his PCP that the lesion was benign; without dermoscopy, his diagnosis of LM would have likely been missed. This case highlights how access to dermoscopy in primary care can be particularly valuable in regions where dermatologists are less accessible and where populations rely on general practitioners for holistic care.

## References

[1] Karponis D, Stratigos IA, Joshy J (2024). Lentigo maligna: a review. Clin Exp Dermatol.

[2] Iznardo H, Garcia-Melendo C, Yélamos O (2020). Lentigo maligna: clinical presentation and appropriate management. Clin Cosmet Investig Dermatol.

[3] Linos E, Li WQ, Han J, Li T, Cho E, Qureshi AA (2017). Lifetime ultraviolet radiation exposure and lentigo maligna melanoma. Br J Dermatol.

[4] Juhász ML, Marmur ES (2015). Reviewing challenges in the diagnosis and treatment of lentigo maligna and lentigo-maligna melanoma. Rare Cancers Ther.

[5] Chen Q, Zheng M, Ling C (2024). Incidence trends of lentigo maligna and lentigo maligna melanoma in the United States from 2000 to 2019. Int J Dermatol.

[6] Vestergaard ME, Macaskill P, Holt PE, Menzies SW (2008). Dermoscopy compared with naked eye examination for the diagnosis of primary melanoma: a meta-analysis of studies performed in a clinical setting. Br J Dermatol.

[7] Mitsaki KS, Apalla Z, Lazaridou E, Lallas K, Lallas A (2024). Risk factors of lentigo maligna as compared to other melanoma subtypes. Int J Dermatol.

[8] Guan LL, Lim HW, Mohammad TF (2021). Sunscreens and photoaging: a review of current literature. Am J Clin Dermatol.

[9] Schwartz RA (2025). Lentigo differential diagnoses. https://emedicine.medscape.com/article/1068503-differential?form=fpf.

[10] Papageorgiou V, Apalla Z, Sotiriou E (2018). The limitations of dermoscopy: false-positive and false-negative tumours. J Eur Acad Dermatol Venereol.

[11] Abbasi NR, Shaw HM, Rigel DS (2004). Early diagnosis of cutaneous melanoma: revisiting the ABCD criteria. JAMA.

[12] Rastrelli M, Tropea S, Rossi CR, Alaibac M (2014). Melanoma: epidemiology, risk factors, pathogenesis, diagnosis and classification. In Vivo.

[13] Kanitakis J (2021). Treatment of lentigo maligna (Review). World Acad Sci J.

[14] Fee JA, McGrady FP, Hart ND (2022). Dermoscopy use in primary care: a qualitative study with general practitioners. BMC Prim Care.

[15] Williams NM, Marghoob AA, Seiverling E, Usatine R, Tsang D, Jaimes N (2020). Perspectives on dermoscopy in the primary care setting. J Am Board Fam Med.

[16] Friche P, Moulis L, Du Thanh A, Dereure O, Duflos C, Carbonnel F (2024). Training family medicine residents in dermoscopy using an e-learning course: pilot interventional study. JMIR Form Res.

[17] Harrison K (2024). The accuracy of skin cancer detection rates with the implementation of dermoscopy among dermatology clinicians: a scoping review. J Clin Aesthet Dermatol.

